# The Dutch patient-based Constant Murley score cannot be used interchangeably with the clinician-based Constant Murley score in shoulder arthroplasty patients

**DOI:** 10.1016/j.jsea.2026.100034

**Published:** 2026-05-05

**Authors:** Brechtje Hesseling, Sander I.B. Perry, Max A. Hoelen, Derek F.P. van Deurzen, Denise Eygendaal, Nina M.C. Mathijssen, Barbara A.M. Snoeker

**Affiliations:** a Reinier Haga Orthopedic Center, Zoetermeer, The Netherlands; b Department of Orthopaedics and Sports Medicine, Erasmus MC, University Medical Center Rotterdam, Rotterdam, The Netherlands; c Department of Orthopedics, Reinier de Graaf Hospital, Delft, The Netherlands; d Department of Epidemiology and Data Science, University Medical Center Amsterdam, University of Amsterdam, Amsterdam, The Netherlands; e Department of Orthopedic Surgery, Shoulder and Elbow Unit, OLVG, Amsterdam, The Netherlands; f Department of Public and Occupational Health, University Medical Center Amsterdam, University of Amsterdam, Amsterdam, The Netherlands; g Department of Clinical Epidemiology and Orthopaedics, Lund University, Lund, Sweden

**Keywords:** Constant Murley score, Shoulder arthroplasty, Agreement, Test-retest reproducibility, Clinimetric study

## Abstract

**Background:**

To find an alternative for the time-consuming Dutch clinician-based Constant Murley score (c-CMS), we assessed its interchangeability with the patient-based CMS (p-CMS) in patients undergoing arthroplasty. Also, we assessed test-retest reproducibility of both CMS versions.

**Methods:**

The English p-CMS was translated into Dutch using forward and backward translation. We administered the c-CMS and p-CMS at 2 time points before surgery and 6 months after surgery. Subsequently, we calculated absolute agreement parameters, both between p-CMS and c-CMS, to study interchangeability. For the repeated measurements of c-CMS and p-CMS, agreement was calculated separately to study test-retest reproducibility.

**Results:**

We recruited 139 patients from 3 different hospitals. Between total p-CMS and total c-CMS, the 95% limits of agreement (LoAs) were −20.4 to 22.6 with a mean bias of 1.1. For test-retest reproducibility of the c-CMS and the p-CMS, LoAs were −15.1 to 15.7 (mean bias 0.3) and −16.6 to 18.8 (mean bias 1.1), respectively. These LoAs were all wider than the shoulder arthroplasty−specific Minimal Clinically Important Differences found in the literature.

**Conclusion:**

The p-CMS cannot be used interchangeably with the c-CMS. In addition, the CMS has become obsolete for quantifying shoulder functionality; shoulder-specific patient-reported outcome measures with better reproducibility are currently available.

To monitor shoulder function in patients with shoulder arthroplasty (SA) and to evaluate the effectiveness of shoulder interventions, the Constant Murley score (CMS)^7^^,^^8^ is widely used in clinical practice and research.^22^ Compared to shoulder-specific patient-reported outcome measures (PROMs), the CMS is characterized by the addition of an objective evaluation of shoulder function through measurements of range of motion and strength.

However, the CMS is time-consuming, taking time away from shared decision-making and patient counseling. In an effort to negate these time constraints, several authors have developed or translated a patient-based CMS (p-CMS) to enable CMS collection outside consultation visits; German,^3^ English,^16^ French,^5^ and Turkish^19^ versions exist. However, the clinician-based CMS (c-CMS) can be replaced by a p-CMS only if they are interchangeable.

Interchangeability of existing p-CMS versions has been studied in various ways. Boehm et al^3^ found the German p-CMS to correlate well with the c-CMS (ρ = 0.82) and concluded that the p-CMS can be used for follow-up of patients after shoulder surgery. Levy et al^16^ created an English p-CMS and found substantial to almost perfect agreement. They concluded that the p-CMS can be used interchangeably with the c-CMS. Chelli et al^5^ designed a French version of the p-CMS and assessed correlation and mean differences. Although patients underestimated their score on average by 3 points compared to clinicians, the authors concluded that the French p-CMS is a good estimator of the c-CMS. Finally, Mısırlıoğlu et al^19^ translated Levy's p-CMS into Turkish and found lower weighted kappa values than Levy et al.^16^ Together with concerns about differences in test protocols and scoring methods, they therefore concluded that the p-CMS cannot be used interchangeably with the c-CMS.

To date, a Dutch patient-based version has not yet been created or translated. Given the mixed results described above, it is prudent to study whether a Dutch p-CMS could be used interchangeably with the c-CMS.

The primary aim of our study was therefore to translate the English p-CMS into Dutch and to assess absolute agreement between the p-CMS and c-CMS versions in patients undergoing SA. The secondary aim was to analyze the test-retest reproducibility of both the Dutch p-CMS and c-CMS.

## Materials and methods

### Study population

This multicenter, prospective cohort study was conducted in 3 hospitals in the Netherlands: Reinier de Graaf Hospital and Haga Hospital (merged in 2020 to the Reinier Haga Orthopedic Centre) and Onze Lieve Vrouwe Gasthuis hospital. From September 2016 to February 2021, patients of the orthopedic surgery departments were screened for eligibility according to the following inclusion criteria: age ≥18 years, scheduled to undergo primary or revision SA (either hemiarthroplasty or anatomic or reversed total SA), good command of the Dutch language, and able and willing to participate. Exclusion criteria were cognitive impairment, arthroplasty for acute fractures, and difficulty with the Dutch language. All participating patients provided written informed consent before the study. The study was reviewed by the regional Medical Ethics Committee, which declared that the study did not fall within the scope of the Medical Research Involving Human Subjects Act (METC Leiden, Den Haag, Delft, in the Netherlands, METC-no. 16-084).

### Constant Murley score

The CMS measures shoulder function using 10 items over 4 domains: pain (15 points), daily activities (20 points), range of motion (40 points), and strength (25 points). The total score ranges from 0 to 100, with higher scores indicating better shoulder function. For the p-CMS, we obtained permission to use and translate the version created by Levy et al.^16^ The translation into Dutch was performed using forward and backward translation by a translation service affiliated with the Dutch Association of Interpreters and Translators. We used the photographs and scoring guidelines from the original English version. The final Dutch version is available in the Appendix.

### Data collection

We prospectively collected patient- and clinician-based CMS at 3 time points: twice preoperatively (ideally spaced 2 weeks apart, T0 and T1) and 6 months postoperatively (T2). During each visit, patients and clinicians were blinded to the clinician- and patient-based scores, respectively.

Clinician-based scores were obtained by 4 orthopedic surgeons, 3 physician assistants or a researcher with a minimum of 10 years of experience as a physiotherapist. All raters were specialized in treating shoulder disorders. We deliberately did not standardize the CMS assessment protocol or provide study-specific training across raters to incorporate existing practice variation.

Most patient-based scores were obtained via digital questionnaires. Paper questionnaires were used in case e-mail was not available to patients. Missing answers or multiple answers to single-answer items were clarified by phone. If a patient could not be reached, we marked the answers as missing (when completely missing in the questionnaire) or selected the worst option (when multiple answers on a single question were given).

In addition to CMS, we collected data on age, gender, pre-operative diagnosis, type of prosthesis, primary or revision arthroplasty, length of hospital stay, and change in shoulder status at T1 and T2 with anchor questions. All data were stored on electronic case report forms using Castor EDC.^4^

### Statistical analysis

All statistical analyses were conducted in R 4.5.0.^23^

We calculated descriptive statistics for our sample. Continuous data are presented as mean with standard deviation and 95% confidence interval (CI) or, in case not normally distributed, as median with interquartile range. Nominal and ordinal data are presented as numbers and percentages.

Similar to Levy et al,^16^ we used the scores from both T0 and T2 for the comparison between the c-CMS and the p-CMS: scores at T0 and T2 from the same patient can be seen as unique measurements due to the surgery in between. This also ensures that we cover the largest possible range of CMS scores. We did not impute missing data.

### Scoring differences between the clinician-based Constant Murley score and patient-based Constant Murley score

As already stated by Mısırlıoğlu et al,^19^ the scoring methods for the c-CMS and p-CMS differ on the items “pain,” “work,” “recreational activities,” and “ability to position hand in space.” We have adapted their suggested modifications to the c-CMS scoring as follows.

For “work” and “recreational activities,” the c-CMS is scored from 1 to 4 points, while the p-CMS is scored at 0, 2, or 4 points. C-CMS scoring was therefore modified by recoding 1 point to 0 points, and 3 points to 2 points.

For “ability to position the hand in space,” the c-CMS allows for 0 points, while the p-CMS starts at 2 points. C-CMS scoring was therefore modified by recoding 0 points to 2 points.

The item “pain” in the c-CMS is scored on a scale of 0 (no), 5 (mild), 10 (moderate), or 15 points (severe). For the p-CMS, “pain” is the average of question a1 (similarly scored to the c-CMS) and question a2 (a numeric rating scale from 0 (no pain) to 15 (maximum pain), of which the inverse is taken). We did not make any modifications to this item, but for the percentage agreement analysis and weighted kappa analysis (described below), it was necessary to use only item a1 of the p-CMS instead of the total item “pain.”

We calculated the total c-CMS with the modifications as well and performed our main analyses on the modified c-CMS scoring. The original scoring of the c-CMS was used in additional analyses.

### Agreement between clinician-based Constant Murley score and patient-based Constant Murley score

To assess the interchangeability of the 2 methods, the most appropriate measure is agreement.^31^^,^^32^ After all, for interchangeability, the most pressing question for the clinician is, “If the patient rates themselves, would they fill in the same answer as I would?.” If the answer is yes, the score is interchangeable. However, if the answer is no, the clinician would need to repeat the measurement themselves.

We studied agreement for each separate item as well as for the total score of both versions. For the ordinal items “pain,” “work,” “recreational activities,” “undisturbed sleep,” “ability to position hand in space,” and the mobility items, we calculated the percentage exact agreement: the percentage of cases in which the patient gave the exact same score as the clinician. For “pain,” only item a1 from the p-CMS was used.

For ordinal items with 5 or 6 answer categories (ie, “ability to position hand in space” and the mobility items), we also calculated the percentage adjacent agreement: the percentage of cases in which the scores were the same or differed by only one level (either up or down).

For the items “strength” and for the total score, we calculated the limits of agreement (LoAs) using the Bland-Altman method.^18^ The LoAs give a range within which 95% of the differences between the measurements are expected to lie, and it is clinically intuitive to interpret.

### Reliability of clinician-based Constant Murley score versus patient-based Constant Murley score

To directly compare the performance of the Dutch p-CMS to the original English p-CMS^16^ and to p-CMS versions in other languages,^19^ we emulated their reliability analyses by calculating weighted kappa for the ordinal items. We only used the modified c-CMS scoring for the items “work,” “recreational activities,” and “ability to position hand in space.” For the item “strength” and the total score, we also calculated the intraclass correlation coefficient (ICC) using a two-way random-effects model, absolute agreement (ICC[2,1]).^14^

### Agreement for test-retest reproducibility of clinician-based Constant Murley score and of patient-based Constant Murley score

To assess the test-retest reproducibility of both the c-CMS and the p-CMS, we repeated the agreement analyses described above on the repeated measurements (ie, T0 and T1) of each instrument. In addition, we calculated the standard error of measurement (SEM_consistency_ = √σ^2^_error_) for the test-retest reproducibility of c-CMS and p-CMS as an indication of the measurement error of a score with respect to the “true” score.^32^^,^^33^ The value of σ^2^_error_ was derived from a two-way random-effects model, consistency (ICC[3,1]).^14^

Finally, we used the SEM to determine the smallest detectable change (SDC) for the c-CMS and p-CMS by calculating SDC = 1.96 x √2 x SEM.^32^

The planned measurement interval between T0 and T1 was 14 days to reduce possible recall bias at T1. However, shoulder function could change within those 14 days. For the test-retest analysis, we therefore added 2 sensitivity analyses: only on patients who answered “a little deterioration,” “no change,” or “a little improvement” on the T1 anchor question (the “less strict” sensitivity analysis), and only on patients who answered “no change” on the T1 anchor question (the “more strict” sensitivity analysis).

### Sample size

The guidelines published by the COnsensus-based Standards for the selection of health Measurement INstruments initiative advocate a sample size of 100 subjects for studies that evaluate measurement properties.^21^^,^^28^ To account for expected loss to follow-up, we therefore aimed to include at least 125 subjects.

## Results

### Study sample

**Figure 1:**
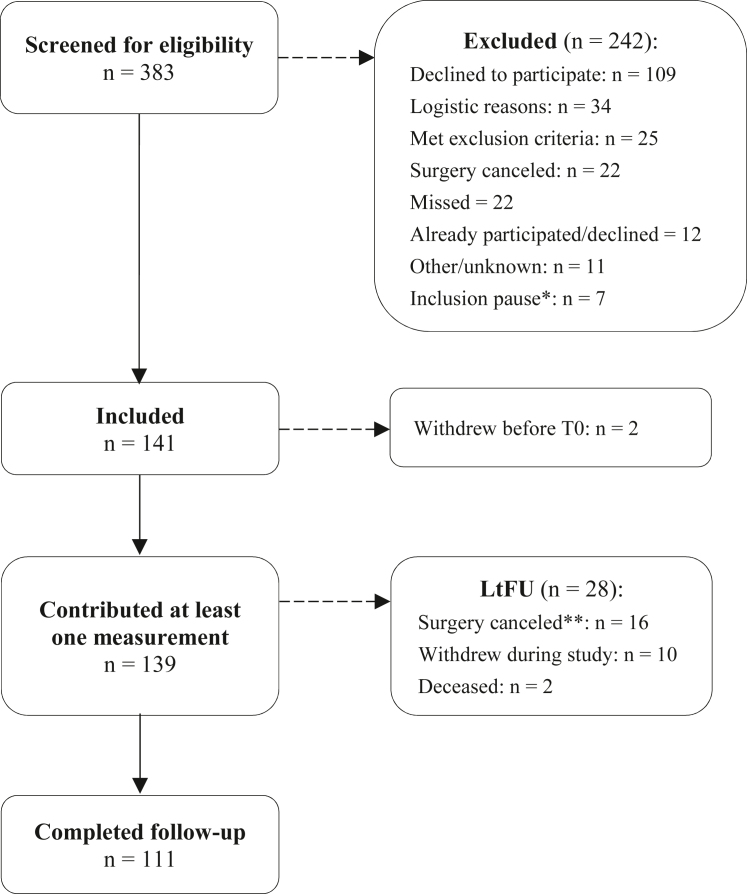
Study flowchart. ∗: The transition from Reinier de Graaf Hospital and Haga Hospital into Reinier Haga Orthopedic Center (RHOC) resulted in an inclusion pause of approximately two months for these centers. ∗∗: When surgery was canceled, measurements at T2 were always missing, but patients could still contribute to T0 and/or T1. *LtFU*, lost to follow-up.

Of the 383 screened patients, 141 provided informed consent and were included in the study. Two subjects withdrew before any measurements were taken, leaving 139 subjects for analysis. See Figure 1 for complete study flow details.

The majority of subjects were female (75.5%), the mean age was 69.7 (standard deviation 9.6) years, and anatomic total shoulder arthroplasty was performed equally often as reverse total shoulder arthroplasty. For more details, see Table I. Details on the distribution of CMS scores and measurement intervals are in Table II.

**Table I:** Patient characteristics *(n = 139)*.

Variable	Entire sample (N = 139)
Gender∗	
Male	34 (24.5%)
Female	105 (75.5%)
Age†	69.7 ± 9.6
Pre-operative diagnosis∗	
Primary osteoarthritis	86 (61.9%)
Cuff tear arthropathy	32 (23.0%)
Avascular necrosis	9 (6.5%)
Secondary osteoarthritis (fracture sequelae)	6 (4.3%)
Massive irreparable cuff rupture	4 (2.9%)
Other	2 (1.4%)
Primary or revision∗	
Primary arthroplasty	135 (97.1%)
Revision arthroplasty	4 (2.9%)
Type of prosthesis∗	
aTSA	56 (40.3%)
rTSA	56 (40.3%)
HA	7 (5.0%)
No surgery	20 (14.4%)

*aTSA*, anatomic total shoulder arthroplasty; *rTSA*, reverse total shoulder arthroplasty; *HA*, hemiarthroplasty.

∗ Values are given as *n* (*%*).

† Values are given as *mean ± standard deviation.*

**Table II:** CMS scores and measurement intervals.

Variable	Entire sample (N = 139)
Clinician CMS∗	
T0	31.3 ± 12.6
T1	31.1 ± 12.5
T2	59.3 ± 16.0
Patient CMS∗	
T0	27.1 ± 13.1
T1	26.2 ± 13.2
T2	59.1 ± 17.3
Intervals†*(in d)*	
Clinician T0 – T1	14 [11-23]
Patient T0 – T1	14 [13-18]
Clinician vs. patient T0	2 [1-6]
Clinician vs. patient T2	2 [1-4]

*CMS*, Constant Murley score.

∗ Values are given as mean ± standard deviation.

† Values are given as median (interquartile range).

### Agreement between clinician-based Constant Murley score and patient-based Constant Murley score

The percentage exact agreement between the c-CMS and the p-CMS ranged from 32% to 67%, with the objective assessment (ie, mobility items) having lower exact agreement than the subjective assessment. The percentage adjacent agreement ranged from 67% to 84%. Table III shows the percentage agreement for each ordinal item.

**Table III:** Agreement within clinician CMS, within patient CMS, and between clinician and patient CMS.

	Clinician CMS vs. patient CMS *n = 224*		Test – retest Clinician CMS *n = 112*	Test – retest Patient CMS *n = 132*
Separate CMS items	Agreement	Κ_w_	Agreement	
Subjective assessment				
Pain‡		0.83 (0.79-0.87)		
Exact agreement	65%		67%	63%
Work		0.57 (0.49-0.64)		
Exact agreement – modified∗	59%			
Exact agreement – original†	35%		71%	77%
Recreational activities		0.41 (0.31-0.52)		
Exact agreement – modified∗	51%			
Exact agreement – original†	24%		64%	75%
Undisturbed sleep		0.62 (0.52-0.72)		
Exact agreement	67%		72%	77%
Ability to position hand in space		0.65 (0.56-0.73)		
Exact agreement – modified∗	43%			
Exact agreement - original†	42%		46%	53%
Adjacent agreement – modified∗	78%			
Adjacent agreement – original†	78%		81%	87%
Objective assessment				
Forward elevation		0.67 (0.59-0.74)		
Exact agreement	35%		49%	52%
Adjacent agreement	84%		89%	88%
Lateral elevation		0.63 (0.55-0.71)		
Exact agreement	37%		65%	56%
Adjacent agreement	83%		93%	89%
Functional external rotation		0.57 (0.47-0.66)		
Exact agreement	32%		54%	53%
Adjacent agreement	67%		85%	80%
Functional internal rotation		0.44 (0.33-0.55)		
Exact agreement	36%		57%	52%
Adjacent agreement	79%		94%	95%
Strength				
ICC		0.970	-	-
SEM_consistency_	-		2.0 lb	1.9 lb
CMS total score				
ICC		0.875∗ 0.874†		
SEM_consistency_			6 points∗^,^†	7 points
SDC			16 points∗^,^†	18 points

*CMS*, Constant Murley score; *ICC*, intraclass correlation coefficient; *Κ*_*w*_ = weighted kappa; *SDC*, smallest detectable change; *SEM*, standard error of measurement.

Agreement is presented as *percentage exact agreement* or *percentage adjacent agreement.*

Reliability is presented as *Κ*_*w*_*(95% CI) or ICC.*

∗ Modified CMS scoring method.

† Original CMS scoring method.

‡ For the p-CMS, only question a1 is used for the percentage agreement and Κ_w_.

**Figure 2:**
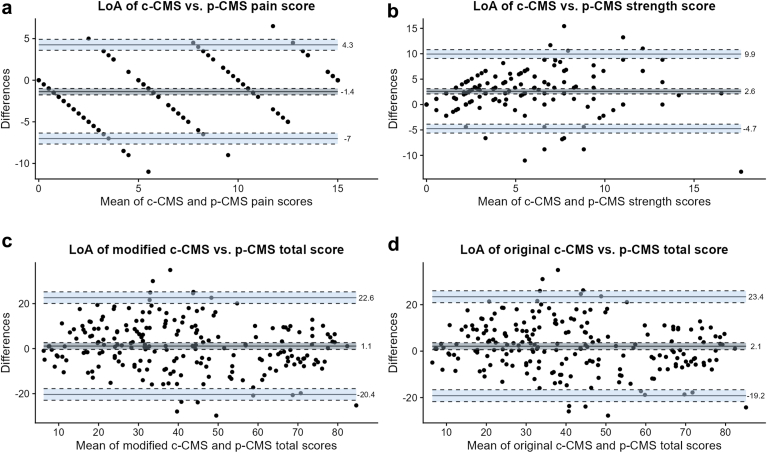
Limits of agreement for the comparison of c-CMS with p-CMS for (**a**) pain score, (**b**) strength score, (**c**) modified c-CMS total score, and (**d**) original c-CMS total score. *c-CMS*, clinician-based Constant Murley score; *p-CMS*, patient-based Constant Murley score; *LoA*, limit of agreement.

In the Bland-Altman analysis, the strength assessment and the total score had a mean bias of 2.6 lbs and 1.1 points, respectively. Figure 2 *A*–*D* show the Bland-Altman plots with corresponding LoAs and 95% CIs. All exact values can be found in Table A-I in the Appendix.

### Reliability of clinician-based Constant Murley score versus patient-based Constant Murley score

Our weighted kappa values were lower than those found by Levy et al,^16^ showing moderate agreement at most. Only the item “pain” showed substantial agreement. The ICCs for strength and total score showed good reliability for these scores. The exact results of our reliability analysis can be found in Table III.

### Test-retest reproducibility of clinician-based Constant Murley score and of patient-based Constant Murley score

**Figure 3:**
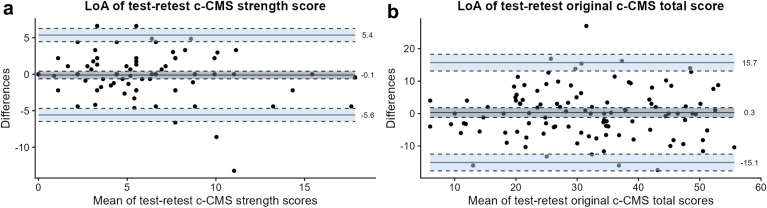
Limits of agreement for test-retest reproducibility of the c-CMS for (**a**) strength score and (**b**) original c-CMS total score. *c-CMS*, clinician-based Constant Murley score; *LoA*, limit of agreement

**Figure 4:**
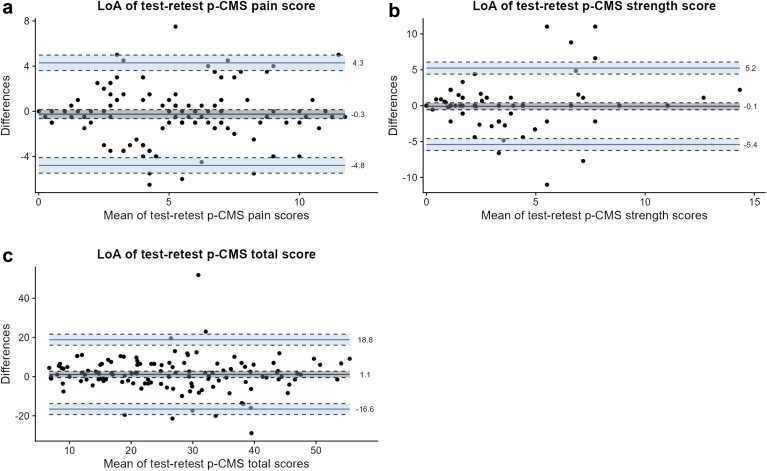
Limits of agreement for test-retest reproducibility of the p-CMS (**a**) pain score, (**b**) strength score, and (**c**) total score. *p-CMS*, patient-based Constant Murley score; *LoA*, limit of agreement

The percentage agreement, SEM, and SDC of both the c-CMS and p-CMS, calculated from the repeated measurements at T0 and T1, are presented in Table III. Figures 3 and 4 show the Bland-Altman plots of both versions. There were no large differences in test-retest reproducibility between the c-CMS and p-CMS. Again, the subjective assessment showed higher agreement than the objective assessment in both CMS versions. For the total score, the mean bias and LoAs were slightly smaller for the c-CMS (Fig. 3*B*) than for the p-CMS (Fig. 4*B*). Exact values of the mean biases, LoAs, and corresponding CIs are presented in Table A-I in the Appendix.

Table IV presents the descriptive statistics for the anchor questions. The planned sensitivity analyses did not substantively change the test-retest reproducibility of the c-CMS or p-CMS, although the LoAs of the total p-CMS narrowed slightly. Sensitivity analysis results are presented in Table A-II and Figures A1-4 in the Appendix.

**Table IV:** Descriptive statistics for anchor questions at T1 and T2.

Self-reported change in shoulder function	T1	T2
Very much deteriorated	5 (3.5%)	-
Much deteriorated	11 (7.8%)	5 (3.5%)
A little deteriorated	51 (36.2%)	4 (2.8%)
Unchanged	63 (44.7%)	4 (2.8%)
A little improved	2 (1.4%)	13 (9.2%)
Much improved	-	46 (32.6%)
Very much improved	-	36 (25.5%)
NA	9 (6.4%)	33 (23.4%)

*NA*, not available.

## Discussion

Our study shows that agreement between the c-CMS and p-CMS in patients undergoing SA is insufficient to warrant direct interchangeability. In addition, the test-retest reproducibility of both the c-CMS and p-CMS is poor, with SDCs exceeding 10% of the 0-100 measurement scale.

First, we address whether the 2 methods are interchangeable by examining the wide LoAs for the total score of the c-CMS compared to the p-CMS. One method can only reliably replace the other if the measurement error (LoA or SDC) is smaller than the Minimal Clinically Important Difference (MCID).^1^^,^^11^^,^^27^ After all, if the measurement error is larger than the MCID, differences in outcomes could be attributed solely to differences in methods, not to true clinically relevant change. In our study, the LoAs for c-CMS versus p-CMS total scores (upper LoA 22.6, lower LoA −20.4) are much wider than the MCIDs of the CMS total score, even when accounting for CIs around the estimated LoAs (see Table A-I in the Appendix); reported MCIDs in SA literature range from −0.3 to 12.8.^13^^,^^25^^,^^34^

This is especially problematic when comparing a new p-CMS measurement from one individual to earlier-obtained c-CMS scores from the same individual. A difference that reaches the MCID may imply a relevant change, whereas in reality it could simply be due to measurement error. The same holds true when comparing p-CMS measurements with c-CMS values from other scientific studies.

We therefore cannot encourage the interchangeable use of the Dutch p-CMS with the c-CMS, even though previous studies concluded that other p-CMS versions performed well enough to warrant interchangeability.

Compared to Levy et al,^16^ our LoAs are much wider. This may be due to differences in populations: Levy et al^16^ included patients with a wide variety of shoulder conditions and surgical procedures, whereas we included only SA patients. More importantly, subjects in Levy et al^16^ had a mean age of 54 years. In our study, the mean age was almost 70 years. Self-assessment may be easier for younger patients or those with other shoulder conditions or surgeries than SA.

In contrast, our results do support those of Mısırlıoğlu et al^19^: we found similar low-weighted kappa values.

For the test-retest reproducibility of the p-CMS, we again found wide LoAs for the p-CMS total score. Just as with the comparison between p-CMS and c-CMS, the LoAs were greater than the aforementioned MCIDs. When performing the sensitivity analyses, the LoAs became narrower and approximated the largest MCID of 12.8 points.^13^ However, in the sensitivity analyses, the SDC remained high at 13 points or more, still above the MCID, indicating poor test-retest reproducibility.

For the test-retest reproducibility of the c-CMS, we found similar poor results: an SDC of at least 16 points and LoAs exceeding the MCIDs. Our LoAs align with the LoAs for the c-CMS reported by other authors,^2^^,^^20^ although our SDC was slightly larger than that reported by Moeller et al^20^ (SDC = 13.1). Given the wide LoAs from our study and previous studies, we argue that the test-retest reproducibility of the c-CMS is suboptimal.

This suboptimal reproducibility of the c-CMS raises concerns about its widespread use in both clinical practice and scientific research and, at the same time, may create an opportunity to use the p-CMS despite its lack of interchangeability with the c-CMS.

On the one hand, one could argue that the c-CMS is of limited value in evaluating change in a patient's shoulder function accurately. Since the LoAs and SDCs encompass SA-specific MCIDs, the question is whether we can actually measure relevant change within patients. Furthermore, the greatest perceived benefit of the c-CMS is the objective evaluation of strength and ROM. However, the benefit is limited if we are not able to reproduce these domains.

On the other hand, despite suboptimal test-retest reproducibility, in practice, the c-CMS remains widely accepted for evaluating shoulder function. Given the comparable reproducibility of the p-CMS to the c-CMS, although poor, one could argue that the p-CMS is similarly acceptable. In that view, the p-CMS could be implemented in standard care to alleviate time constraints during consultations by having patients complete the p-CMS beforehand. In the context of scientific studies, the p-CMS could eliminate logistical barriers to collect mobility and strength data by allowing patients to complete it remotely. However, it is essential to acknowledge that using the p-CMS would have severe limitations, as described in the section above on interchangeability. The potential use of the p-CMS comes with a serious trade-off.

### Recommendations on the use of the clinician-based Constant Murley score and patient-based Constant Murley score

As stated above, we cannot encourage the interchangeable use of the c-CMS and the Dutch p-CMS. In addition, we strongly urge the orthopedic community to critically consider whether the benefit of the objective evaluation in the CMS actually outweighs its drawbacks, given its poor reproducibility. More specifically, we argue that including range of motion and strength is no longer of major benefit.

Range of motion and strength are primarily proxies for assessing whether patients can use their shoulders for daily tasks. This approach was especially valuable when the CMS was introduced in 1987,^7^ at a time when outcome evaluations focused chiefly on clinical measures such as prosthesis survival and range of motion.^30^ However, over the last decades, the use of PROMs has increased markedly^12^^,^^15^ because they focus on outcomes that matter to patients (ranging from joint-specific symptoms to functional limitations and health-related quality of life).^6^

Many shoulder-specific PROMs currently in use, such as the Oxford Shoulder Score^9^ and the Simple Shoulder Test^17^, include questions about functional activities that require a certain range of motion and strength. Although studies assessing agreement parameters for these PROMs are scarce (most studies use relative reliability measures), reproducibility appears better for these PROMs than for the c-CMS.^10^^,^^26^^,^^29^ Given the poor reproducibility of the c-CMS, we argue that it has become obsolete. Consequently, we likewise do not advocate the use of the Dutch p-CMS over existing shoulder-specific PROMs, either.

## Strengths and limitations

Just as every other study, ours has both strengths and limitations.

The c-CMS protocol was not standardized for the assessors in this study. We purposefully chose not to standardize to match clinical practice; Rocourt et al^24^ noted large discrepancies in how test protocols were defined and performed in different European hospitals.

On the other hand, Blonna et al^2^ found higher reliability and reproducibility with a standardized version of the CMS than with a non-standardized version. Although the lack of standardization could thus have reduced our agreement results for both interchangeability and test-retest outcomes, our results are representative of use in clinical practice and in studies that use c-CMS scores collected in standard care.

A major strength of our study is the use of absolute agreement parameters instead of relative reliability parameters. Agreement and reliability parameters have different uses and interpretations.^31^^,^^32^ Agreement assesses how close scores for repeated measurements are (both for intrarater and interrater agreement) and thus concerns measurement error in isolation. Conversely, reliability assesses how well patients can be distinguished from one another despite measurement error; this error is thereby related to the variability within a sample. The conceptual differences between agreement and reliability explain why the ICC and weighted kappa (relative reliability parameters) can be high while absolute agreement is poor: in such cases, the variability between persons is large relative to the measurement error. The conceptual differences also mean that reliability parameters are suited for instruments with discriminate purposes, whereas agreement parameters are suited for instruments with evaluative purposes. The CMS is mainly used to evaluate changes in shoulder function; it is not used discriminatively to, for example, decide which patients need surgery. Subsequently, absolute agreement parameters are the most appropriate measures for assessing interchangeability and reproducibility of the c-CMS and p-CMS.

## Conclusion

The Dutch p-CMS cannot be used interchangeably with the c-CMS. In addition, we argue that the CMS has become obsolete for quantifying shoulder functionality due to the availability of many shoulder-specific PROMs.

## Disclaimers:

Funding: This work was supported by the Scientific Committee of the Reinier de Graaf Hospital, Delft, the Netherlands (grant no. 621608). The funding source was not involved in the study design, collection analysis, and interpretation of the data, writing of the report and decision to submit the article for publication.

Conflicts of interest: The authors, their immediate families, and any research foundations with which they are affiliated have not received any financial payments or other benefits from any commercial entity related to the subject of this article.

## References

[1] Barten J.A., Pisters M.F., Huisman P.A., Takken T., Veenhof C. (2012). Measurement properties of patient-specific instruments measuring physical function. J Clin Epidemiol.

[2] Blonna D., Scelsi M., Marini E., Bellato E., Tellini A., Rossi R. (2012). Can we improve the reliability of the Constant-Murley score?. J Shoulder Elbow Surg.

[3] Boehm D., Wollmerstedt N., Doesch M., Handwerker M., Mehling E., Gohlke F. (2004). [Development of a questionnaire based on the constant-murley-score for self-evaluation of shoulder function by patients]. Unfallchirurg.

[4] Castor EDC (2019). Castor electronic data capture. https://castoredc.com.

[5] Chelli M., Levy Y., Lavoué V., Clowez G., Gonzalez J.F., Boileau P. (2019). The "Auto-Constant": can we estimate the Constant-Murley score with a self-administered questionnaire? A pilot study. Orthop Traumatol Surg Res.

[6] Ciani O., Federici C.B. (2020). Value lies in the eye of the patients: the why, what, and how of patient-reported outcomes measures. Clin Ther.

[7] Constant C., Murley A. (1987). A clinical method of functional assessment of the shoulder. Clin Orthop Relat Res.

[8] Constant C.R., Gerber C., Emery R.J., Søjbjerg J.O., Gohlke F., Boileau P. (2008). A review of the Constant score: modifications and guidelines for its use. J Shoulder Elbow Surg.

[9] Dawson J., Fitzpatrick R., Carr A. (1996). Questionnaire on the perceptions of patients about shoulder surgery. J Bone Joint Surg Br.

[10] Frich L.H., Noergaard P.M., Brorson S. (2011). Validation of the Danish version of Oxford Shoulder Score. Dan Med Bull.

[11] Grunow J.J., Hartmann L., Ulm B., Maechler M., Blobner M., Seidenspinner K. (2025). Reliability of pre-admission patient-reported outcome measures postoperatively assessed via proxies: a prospective, multicenter observational study. Crit Care.

[12] Jin H., He M., Xie W., Xiong Z., Deng Z., Li Y. (2023). Research trends of patient-reported outcome measures in orthopedic medical practices: a bibliometric and visualized Study. Medicina (Kaunas).

[13] Kolin D.A., Moverman M.A., Pagani N.R., Puzzitiello R.N., Dubin J., Menendez M.E. (2022). Substantial inconsistency and variability exists among minimum clinically important differences for shoulder arthroplasty outcomes: a systematic review. Clin Orthop Relat Res.

[14] Koo T.K., Li M.Y. (2016). A guideline of selecting and reporting intraclass correlation coeffici ents for reliability research. J Chiropr Med.

[15] Lakey E., Hunt K.J. (2019). Patient-Reported outcomes in foot and ankle orthopedics. Foot Ankle Orthop.

[16] Levy O., Haddo O., Massoud S., Mullett H., Atoun E. (2014). A patient-derived Constant-Murley score is comparable to a clinician-derived score. Clin Orthop Relat Res.

[17] Lippitt S., Matsen F., Fu F., Hawkins R. (1993).

[18] Martin Bland J., Altman D. (1986). Statistical methods for assessing agreement between two methods of cli nical measurement. Lancet.

[19] Mısırlıoğlu T., Eren İ., Taşkıran Ö., Günerbüyük C., Birsel O., Canbulat N. (2022). Turkish version of the patient-based Constant-Murley Score: its cross- cultural adaptation, validity, reliability and comparison with the cli nician-based version. Turk J Phys Med Rehabil.

[20] Moeller A.D., Thorsen R.R., Torabi T.P., Bjoerkman A.-S.D., Christensen E.H., Maribo T. (2014). The Danish version of the modified Constant-Murley shoulder score: rel iability, agreement, and construct validity. J Orthop Sports Phys Ther.

[21] Mokkink L.B., Terwee C.B., Patrick D.L., Alonso J., Stratford P.W., Knol D.L. (2010). The COSMIN checklist for assessing the methodological quality of studies on measurement properties of health status measurement instruments: an international Delphi study. Qual Life Res.

[22] Mosher Z.A., Ewing M.A., Collins C.S., Young P.G., Brabston E.W., Momaya A.M. (2020). Usage trends of patient-reported outcome measures in shoulder literature. J Am Acad Orthop Surg.

[23] Core Team R. (2025).

[24] Rocourt M.H.H., Radlinger L., Kalberer F., Sanavi S., Schmid N.S., Leunig M. (2008). Evaluation of intratester and intertester reliability of the Constant- Murley shoulder assessment. J Shoulder Elbow Surg.

[25] Simovitch R., Flurin P.-H., Wright T., Zuckerman J.D., Roche C.P. (2018). Quantifying success after total shoulder arthroplasty: the minimal clinically important difference. J Shoulder Elbow Surg.

[26] St-Pierre C., Desmeules F., Dionne C.E., Frémont P., MacDermid J.C., Roy J.S. (2016). Psychometric properties of self-reported questionnaires for the evaluation of symptoms and functional limitations in individuals with rotator cuff disorders: a systematic review. Disabil Rehabil.

[27] Terwee C.B., Bot S.D., de Boer M.R., van der Windt D.A., Knol D.L., Dekker J. (2007). Quality criteria were proposed for measurement properties of health status questionnaires. J Clin Epidemiol.

[28] Terwee C.B., Mokkink L.B., Knol D.L., Ostelo R.W., Bouter L.M., de Vet H.C. (2012). Rating the methodological quality in systematic reviews of studies on measurement properties: a scoring system for the COSMIN checklist. Qual Life Res.

[29] van Kampen D.A., Willems W.J., van Beers L.W., Castelein R.M., Scholtes V.A., Terwee C.B. (2013). Determination and comparison of the smallest detectable change (SDC) and the minimal important change (MIC) of four-shoulder patient-reported outcome measures (PROMs). J Orthop Surg Res.

[30] Van Lieshout E.M.M., Wijffels M.M.E. (2020). Patient-reported outcomes: which ones are most relevant?. Injury.

[31] Vet H.C.W., Mokkink L.B., Terwee C.B., Hoekstra O.S., Knol D.L. (2013). Clinicians are right not to like Cohen's κ. BMJ.

[32] Vet H.C.W., Terwee C.B., Knol D.L., Bouter L.M. (2006). When to use agreement versus reliability measures. J Clin Epidemiol.

[33] Weir J.P. (2005). Quantifying test-retest reliability using the intraclass correlation c oefficient and the SEM. J Strength Cond Res.

[34] Zhou A., Xu S., Yew K.S.A., Lie D.T.T. (2023). Minimal clinically important differences for Oxford, constant, and University of California Los Angeles shoulder scores after reverse shoulder arthroplasty to allow interpretation of patient-reported outcome measures and future statistical power analyses. Arthroscopy.

