# Supplementary material for: The Dutch patient-based Constant Murley score cannot be used interchangeably with the clinician-based Constant Murley score in shoulder arthroplasty patients
Source: J Shoulder Elb Arthroplast. 2026 May 5;10(3):100034. doi: 10.1016/j.jsea.2026.100034 (PMC13264057; doi:10.1016/j.jsea.2026.100034)
Supplement: Appendix [file mmc1.pdf]

Appendix Table A-I. Limits of Agreement

Table A-I. Limits of Agreement within c-CMS, within p-CMS, and between c-CMS and p-CMS

	Clinician CMS vs. Patient CMS	Test – retest clinician CMS			Test – retest patient CMS		
		Main analysis	Sensitivity analysis *(less strict)*	Sensitivity analyses *(more strict)*	Main analysis	Sensitivity analysis *(less strict)*	Sensitivity analyses *(more strict)*
Strength *(in lb)*							
Bias	2.6 (2.1 – 3.1)	-0.1 (-0.6 – 0.4)	-0.3 (-0.8 – 0.3)	-0.01 (-0.8 – 0.7)	-0.1 (-0.6 – 0.4)	-0.1 (-0.6 – 0.4)	-0.2 (-0.8 – 0.4)
Upper LoA	9.9 (9.1 – 10.8)	5.4 (4.5 – 6.3)	5.4 (4.4 – 6.4)	5.2 (3.9 – 6.5)	5.2 (4.4 – 6.1)	5.4 (4.4 – 6.3)	4.1 (3.1 – 5.1)
Lower LoA	-4.7 (-5.6 – -3.9)	-5.6 (-6.5 – -4.7)	-5.9 (-6.9 – -4.9)	-5.3 (-6.6 – -4.0)	-5.4 (-6.2 – -4.6)	-5.6 (-6.5 – -4.7)	-4.5 (-5.5 – -3.5)
CMS total score *(in points)*							
Bias	1.1 (-0.3 – 2.6)* 2.1 (0.6 – 3.6)**	0.3 (-1.2 – 1.8)	0.3 (-1.3 – 1.9)	0.2 (-2.2 – 2.6)	1.1 (-0.5 – 2.7)	0.4 (-1.1 – 1.9)	1.2 (-0.5 – 2.8)
Upper LoA	22.6 (20.1 – 25.2)* 23.4 (20.9 – 26.0)**	15.7 (13.1 – 18.3)	16.1 (13.3 – 18.9)	16.6 (12.5 – 20.8)	18.8 (16.1 – 21.6)	16.3 (13.6 – 18.9)	13.5 (10.7 – 16.5)
Lower LoA	-20.4 (-22.9 – -17.8)* -19.2 (-21.7 – -16.7)**	-15.1 (-17.6 – -12.5)	-15.5 (-18.3 – -12.7)	-16.2 (-20.3 – -12.1)	-16.6 (-19.3 – -13.8)	-15.5 (-18.1 – -12.8)	-11.2 (-14.2 – -8.3)

CMS = Constant Murley score, c-CMS = clinician CMS, p-CMS = patient CMS, LoA = Limits of Agreement
Values are given as mean (95% CI)
* = modified CMS scoring method
** = original CMS scoring method. For the test-retest reproducibility, only the original score was used.

Appendix Table A-II. Sensitivity analyses for test-retest reproducibility

Table A-II. Sensitivity analyses for test-retest reproducibility

	Test-retest clinician CMS			Test-retest patient CMS		
	Main analysis	Sensitivity analysis *(less strict)*	Sensitivity analyses *(more strict)*	Main analysis	Sensitivity analysis *(less strict)*	Sensitivity analyses *(more strict)*
**Separate CMS items**						
Subjective assessment						
Pain						
Exact agreement	67%	66%	72%	63%	64%	56%
Work						
Exact agreement	71%	68%	62%	77%	76%	81%
Recreational activities						
Exact agreement	64%	62%	58%	75%	75%	76%
Undisturbed sleep						
Exact agreement	72%	72%	72%	77%	79%	81%
Ability to position hand in space						
Exact agreement	46%	41%	50%	53%	53%	57%
Adjacent agreement	81%	79%	80%	87%	88%	89%
Objective assessment						
Forward elevation						
Exact agreement	49%	51%	45%	52%	51%	57%
Adjacent agreement	89%	88%	80%	88%	90%	94%
Lateral elevation						
Exact agreement	65%	66%	65%	56%	56%	67%
Adjacent agreement	93%	92%	90%	89%	90%	95%
Functional external rotation						
Exact agreement	54%	52%	58%	53%	52%	49%
Adjacent agreement	85%	83%	87%	80%	79%	79%
Functional internal rotation						
Exact agreement	57%	59%	60%	52%	51%	56%
Adjacent agreement	94%	95%	94%	95%	96%	95%
Strength						
$SEM_{consistency}$	2.0 lb	2.0 lb	1.9 lb	1.9 lb	2.0 lb	1.5 lb
**CMS total score**				–		
$SEM_{consistency}$	6 points	6 points	6 points	7 points	6 points	5 points
SDC	16 points	16 points	17 points	18 points	16 points	13 points

CMS = Constant Murley score, SEM = Standard Error of Measurement, SDC = Smallest Detectable Change

Sensitivity analyses – Limits of Agreement for c-CMS test-retest reproducibility

Figure A-1. Less strict sensitivity analysis of test-retest LoA for c-CMS strength and total scores

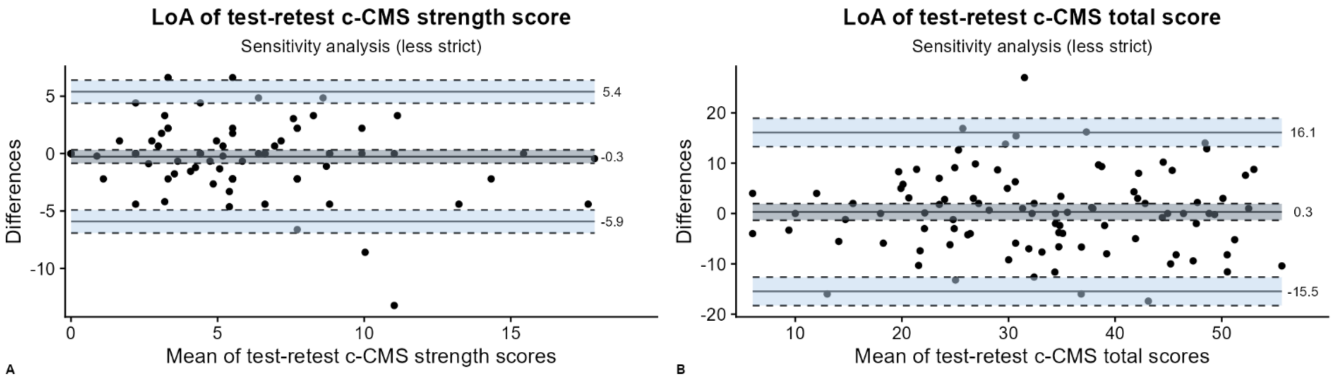

Figure A-2. More strict sensitivity analysis of test-retest LoA for c-CMS strength and total scores

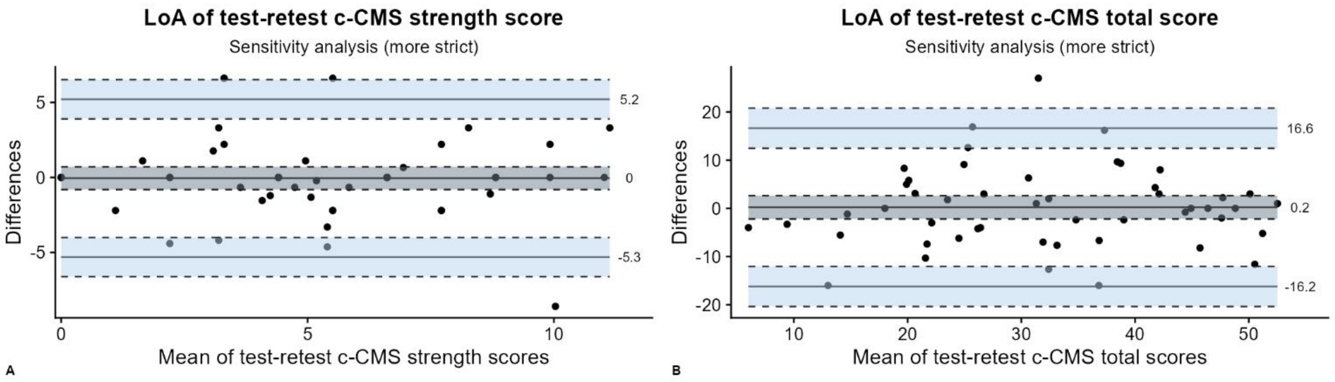

Sensitivity analyses – Limits of Agreement for p-CMS test-retest reproducibility

Figure A-3. Less strict sensitivity analysis of test-retest LoA for p-CMS pain, strength and total scores

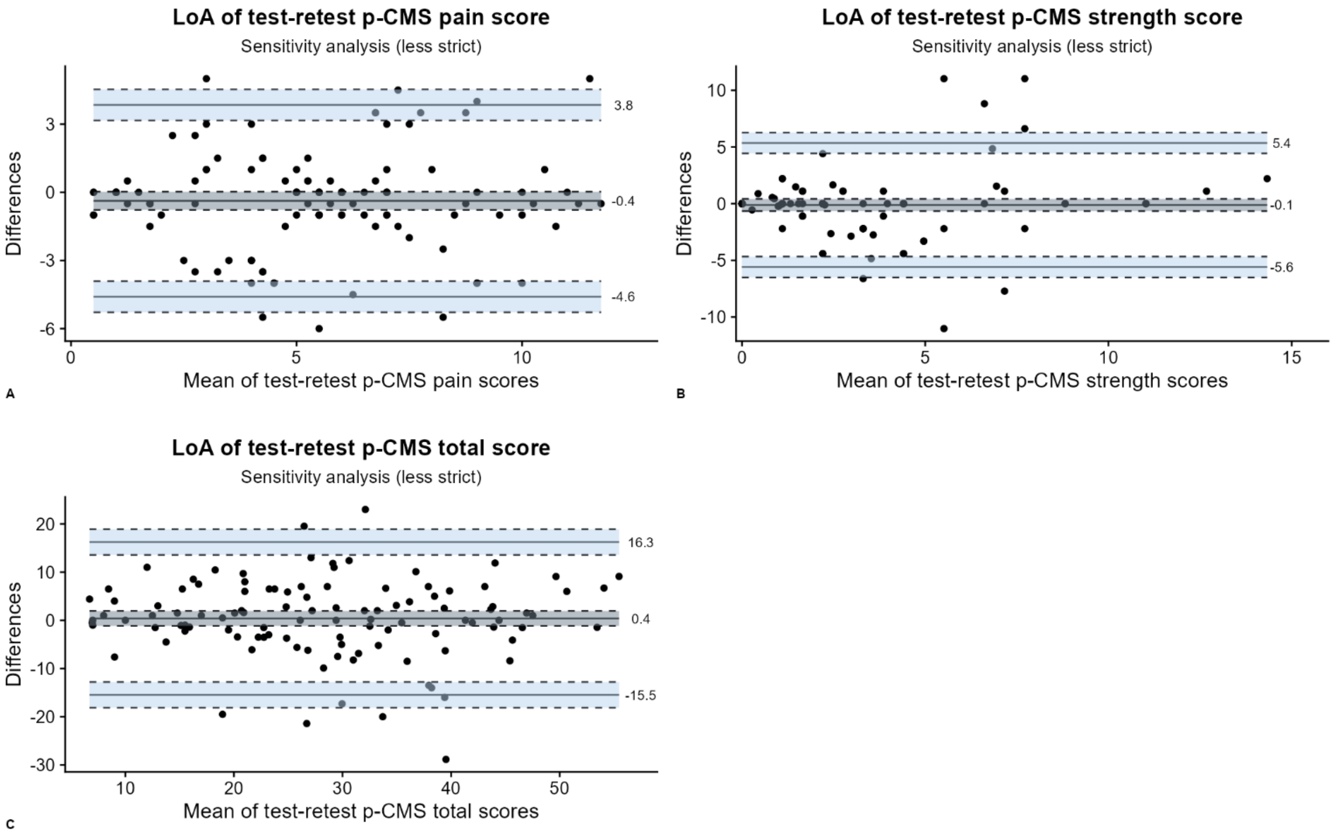

Figure A-4. More strict sensitivity analysis of test-retest LoA for p-CMS pain, strength and total scores

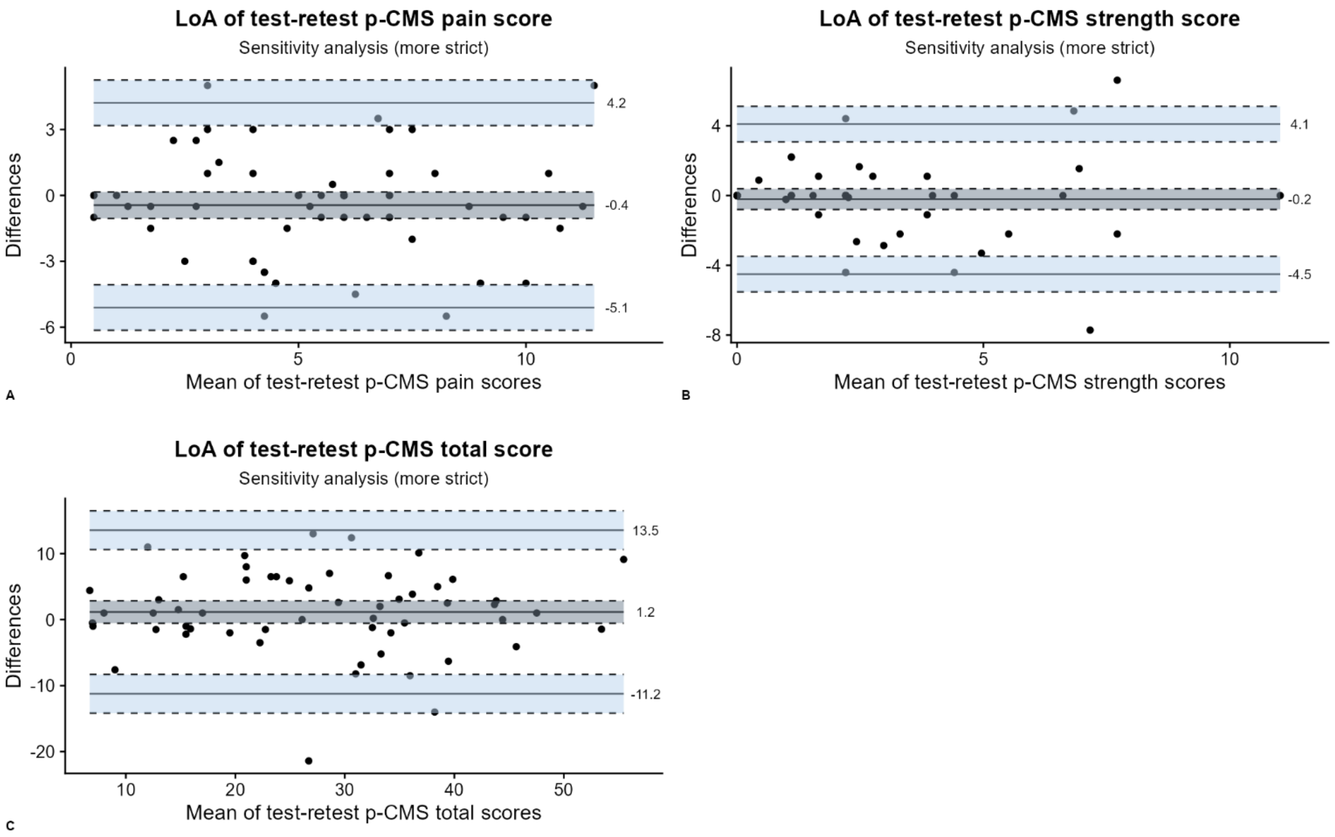

reinier haga orthopedisch centrum

Patiënt-versie Constant-Murley score

T0 – Eerste meetmoment

In te vullen door de onderzoeker

Studienummer

Zijde: rechts / links (doorhalen wat niet van toepassing is)

Datum van invullen: ______________________________

Deze vragenlijst gaat over hoe soepel en sterk uw schouder is, en in welke mate u klachten aan uw schouder ervaart. Lees de vragen en instructies goed door. Kies bij elke vraag het beste antwoord. Het is voor ons erg belangrijk dat u *alle* vragen invult. Wanneer u twijfelt, vul dan het antwoord in dat het best overeenkomt met uw ideale antwoord.

A. Pijn

A1. **Heeft u pijn in uw schouder tijdens normale activiteiten?** (Omcirkel het antwoord dat het meest van toepassing is)

1. GEEN PIJN 2. LICHTE PIJN 3. MATIGE PIJN 4. ERNSTIGE PIJN

A2. **INTENSITEIT VAN DE PIJN**

Indien 0 geen pijn betekent en 15 de ergste pijn is die u kunt verdragen, omcirkelt u het nummer dat de pijn in uw schouder beschrijft wanneer u normale werkzaamheden uitvoert.

☺ 0 1 2 3 4 5 6 7 8 9 10 11 12 13 14 15 ☹

GEEN LICHT MATIG ERNSTIG ONDRAAGLIJK

B. Functie (Omcirkel het antwoord dat het meest van toepassing is)

B1. **Belemmert uw schouder u bij het doen van uw werk of bij het dagelijks leven?**

1. NEE OF EEN BEETJE 2. MATIGE BELEMMERING 3. ERNSTIGE BELEMMERING

B2. **Worden uw vrije tijd en recreatieve activiteiten belemmerd door uw schouder?**

1. NEE OF EEN BEETJE 2. MATIGE BELEMMERING 3. ERNSTIGE BELEMMERING

B3. **Belemmert uw schouder uw nachtrust?**

1. NEE 2. SOMS 3. JA

B4. **Tot welke hoogte kunt u uw arm redelijk pijnloos gebruiken?**

1. MIDDEL 2. BORST 3. NEK 4. OOR 5. BOVEN HET HOOFD

C. Bewegingsbereik

Doe de bewegingen na die het model (op de foto's) uitvoert– het kan handig zijn om deze oefeningen voor een spiegel te doen.

Ga van links naar rechts in iedere rij

- **<u>Vink het hokje aan</u>** (onder de foto) als u **<u>in staat bent</u>** om de beweging uit te voeren
- **<u>Laat het hokje leeg</u>** als u de beweging **<u>niet</u>** kunt uitvoeren

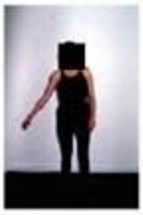
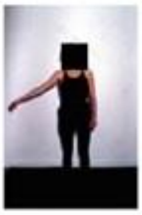
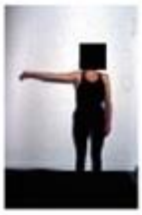
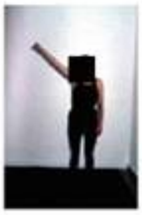
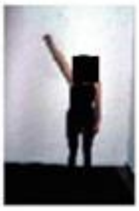
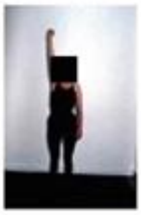

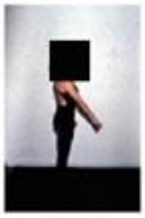
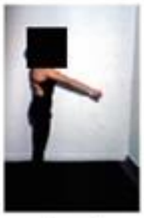
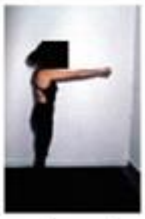
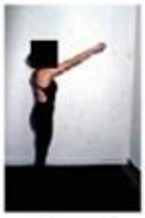
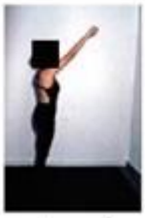
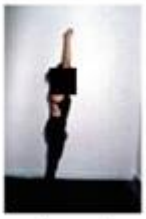

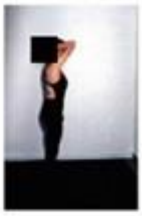
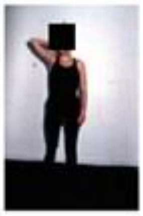
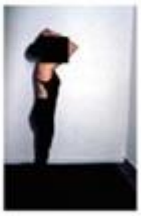
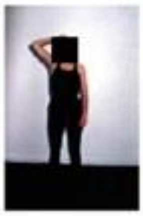
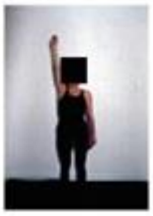

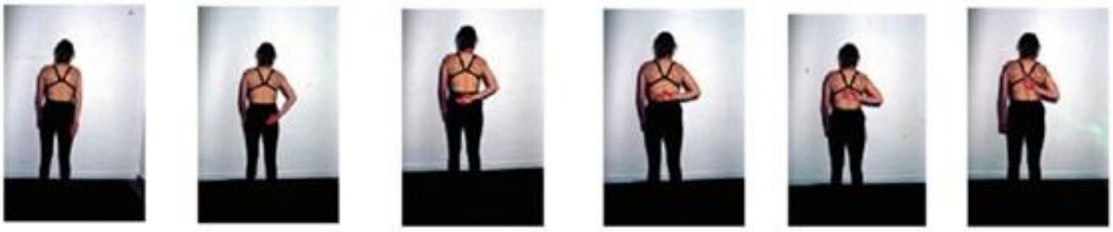

F. Kracht

Bekijk de twee foto's hieronder. Probeer om uw arm drie seconden lang in deze positie te houden, iedere keer met gewichten in de plastic tas.

Gebruik de volgende gewichten in de plastic tas:

- **pakken suiker**

  en / of

- **Gevulde plastic waterflesjes**

  en / of

- **Wat u maar kunt vinden met een bekend gewicht**

Geef in de ruimtes hieronder voor iedere arm aan wat het **maximale gewicht** in kilogrammen of liters was, dat u **3 seconden lang** in deze positie kon dragen.

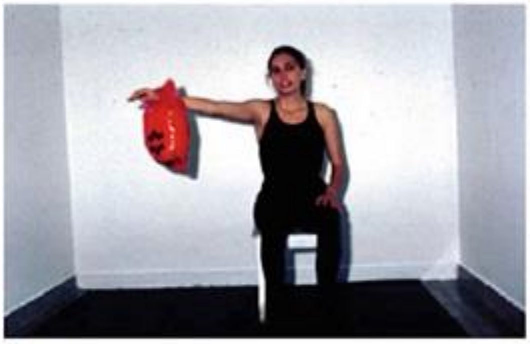

Vooraanzicht

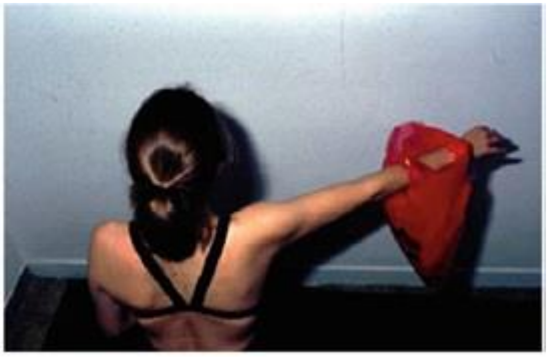

Bovenaanzicht

Het maximale gewicht dat ik in deze positie 3 seconden lang kon dragen was:

Rechter arm: kilogram/liter Linkerarm: kilogram/liter

Deze vragenlijst is vertaald en gepubliceerd met toestemming van The Reading Shoulder Unit, Reading, Berkshire, Verenigd Koninkrijk.
